# Genetic Loci Associated With Fluoride Resistance in *Streptococcus mutans*

**DOI:** 10.3389/fmicb.2018.03093

**Published:** 2018-12-11

**Authors:** Ying Liao, Jingmei Yang, Bernd W. Brandt, Jiyao Li, Wim Crielaard, Cor van Loveren, Dong Mei Deng

**Affiliations:** ^1^West China College of Stomatology, Sichuan University, Chengdu, China; ^2^Nanjing Stomatological Hospital, Nanjing University Medical School, Nanjing, China; ^3^Department of Preventive Dentistry, Academic Center for Dentistry Amsterdam, Vrije Universiteit Amsterdam – University of Amsterdam, Amsterdam, Netherlands; ^4^Guangdong Provincial Key Laboratory of Stomatology, Sun Yat-sen University, Guangzhou, China

**Keywords:** fluoride, *Streptococcus mutans*, resistance, caries prevention, glycolysis, single nucleotide polymorphism

## Abstract

The prolonged exposure of the cariogenic bacterial species *Streptococcus mutans* to high concentrations of fluoride leads to the development of fluoride resistance in this species. Previous studies confirmed the involvement of a mutation in a single chromosomal region in the occurrence of fluoride resistance. The involvement of multiple genomic mutations has not been verified. The aim of this study is to identify multiple genetic loci associated with fluoride resistance in *S. mutans*. The previously published whole genome sequences of two fluoride-resistant *S. mutans* strains (UA159-FR and C180-2FR) and their corresponding wild-type strains (UA159 and C180-2) were analyzed to locate shared chromosomal mutations in fluoride-resistant strains. Both fluoride-resistant strains were isolated in laboratory by culturing their mother strains in media with high concentrations of fluoride. The corresponding gene expression and enzyme activities were accordingly validated. Mutations were identified in two glycolytic enzymes, namely pyruvate kinase and enolase. Pyruvate kinase was deactivated in fluoride-resistant strain C180-2FR. Enolase was less inhibited by fluoride in fluoride-resistant strain UA159-FR than in its wild-type strain. Mutations in the promoter *mutp* constitutively increased the promoter activity and up-regulated the expression of the downstream fluoride antiporters in fluoride-resistant strains. Mutations in the intergenic region *glpFp* led to lower expression of *glpF*, encoding a glycerol uptake facilitator protein, in fluoride-resistant strains than in wild-type strains. Our results revealed that there is overlap of chromosomal regions with mutations among different fluoride-resistant *S. mutans* strains. They provide novel candidates for the study of the mechanisms of fluoride resistance.

## Introduction

Fluoride has been applied for over 50 years as an effective anti-caries agent. Fluoride ions (F^-^) can prevent dental caries through their ability to protect dental hard tissue, as well as to inhibit bacterial growth and metabolism. Its antimicrobial effect is an important part of its caries-preventive mechanism (Ten Cate, 2004). Application of fluoride has been related to decreased number of cariogenic bacterial species, including *Streptococcus mutans* (Yoshihara et al., 2001). When present in bacterial cells, F^-^ can directly or indirectly inhibit metabolic enzymes including enolase, F-ATPase and urease, leading to decreased growth and metabolism (Curran et al., 1994; Guha-Chowdhury et al., 1997; Burne and Marquis, 2000; van Loveren et al., 2008; Pandit et al., 2013). Fluoride-resistant bacteria can survive and grow with fluoride ranging from 400 to 1,000 ppm (21.1–52.6 mM) in the environment (Brussock and Kral, 1987; van Loveren et al., 1993; Liao et al., 2017). Although the fluoride concentrations in oral hygiene products vary from 250 ppm (13 mM) to 1500 ppm (79 mM), the remaining fluoride concentration in dental biofilms after the application of these products was reported to be just between 1.14 ppm (0.06 mM) and 5.7 ppm F (0.3 mM) (Duckworth et al., 1987; Naumova et al., 2012). Therefore, the daily application of fluoride unlikely imposes selective pressure on a healthy oral ecosystem. The results of our recent study indicated, however, that the biofilm fluoride-level may induce resistance in bacteria when the pH of the environment remains low for an extended period of time (Cai et al., 2017).

Oral fluoride-resistant bacterial strains have been isolated both from clinics and laboratories (Hamilton, 1969; Streckfuss et al., 1980; Van Loveren et al., 1991; Sheng and Liu, 2000). Among them, fluoride-resistant *S. mutans* strains have been of special interest because of the well-established role of *S. mutans* in cariogenesis (Lemos et al., 2013). A previous study on the phenotype of fluoride-resistant *S. mutans* suggested that the caries-preventive effect of fluoride may be compromised by the existence of such resistant bacteria (Hoelscher and Hudson, 1996).

Both transient and stable fluoride-resistant *S. mutans* strains have been reported in previous studies (Hamilton, 1969; Brown et al., 1980; Streckfuss et al., 1980; Van Loveren et al., 1991; Sheng and Liu, 2000). The resistant strains isolated from xerostomia patients with long-term use of fluoride gel only possessed transient fluoride resistance (Brown et al., 1980; Streckfuss et al., 1980). Laboratory-derived fluoride-resistant *S. mutans* strains usually possess stable fluoride resistance, which remains for at least 50 generations in absence of fluoride (Van Loveren et al., 1989; Hoelscher and Hudson, 1996; Zhu et al., 2012). Recent studies (Mitsuhata et al., 2014; Liao et al., 2015, 2016) revealed that, while transient resistance might be result of environmental adaptation (Streckfuss et al., 1980), stable resistance is likely the result of chromosomal mutations. Up till now only one chromosomal region has been linked to fluoride resistance, namely the promoter region of fluoride antiporter-coding genes (Mitsuhata et al., 2014; Liao et al., 2015, 2016). Fluoride antiporters can export fluoride ions (F^-^) while importing protons (H^+^) and hence reduce intracellular fluoride concentration (Baker et al., 2012). Knock out studies have shown that the deletion of these fluoride antiporters increased the sensitivity of *S. mutans* to fluoride (Men et al., 2016; Murata and Hanada, 2016). Previously, we discovered that the fluoride-sensitive *S. mutans* strain UA159 became fluoride-resistant when a mutation was introduced in the promoter of the fluoride antiporters. This resistance was obtained through up-regulation of the expression of fluoride antiporters (Liao et al., 2015, 2016).

Besides the mutation in the promoter of fluoride antiporter-coding genes, fluoride resistance may be related to other chromosomal alterations. Brussock and Kral (1987) isolated fluoride-resistant strains in two steps. The second-step isolates, with higher mutation rate, showed stronger resistance than the first-step ones. They thus proposed that multiple genes were involved in fluoride resistance (Brussock and Kral, 1987). Another evidence for the involvement of multiple mutations in fluoride resistance is provided by our previous studies. The naturally selected fluoride-resistant strain which harbor multiple mutations showed stronger resistance to fluoride when compared to the genetically modified strain, which has only one mutation (Liao et al., 2015, 2016). In the fluoride-resistant strain C180-2FR, we have identified 8 single nucleotide polymorphisms (SNPs), including the mutation in the promoter of fluoride antiporters (Liao et al., 2015). These SNPs locate either in the promoter region of functional genes or inside a gene. Although it is unclear whether some or all of the affected loci play a role in fluoride resistance, the abovementioned studies (Brussock and Kral, 1987; Liao et al., 2015, 2016) suggested that fluoride resistance may be acquired through mutations in multiple individual genes, apart or in combination.

In 2013, the full genome sequence of the laboratory-isolated fluoride-resistant strain UA159-FR, derived from strain UA159 (Ajdic et al., 2002), was published in the NCBI database. The aim of this study is to investigate genetic loci associated with fluoride resistance of *S. mutans*. To this end, we compared the previously published genomic sequences of *S. mutans* UA159 and UA159-FR to identify chromosomal mutations in UA159-FR. These mutations were then compared with those in *S. mutans* C180-2FR (Liao et al., 2015) to identify shared regions with mutations in two different fluoride-resistant strains. Furthermore, with bioinformatics prediction (SIFT) and biological evaluation, we prioritized these shared mutations.

## Materials and Methods

### Bacterial Strains and Growth Conditions

The strains used in this study were *S. mutans* UA159, C180-2 and their derived fluoride-resistant strains UA159-FR (kindly provided by Prof. Zhimin Zhang from Jilin University, Changchun, China) and C180-2FR (Van Loveren et al., 1989). Both fluoride-resistant strains were obtained through a step-wise procedure in laboratory (Van Loveren et al., 1989; Zhu et al., 2012). Briefly, the wild-type strains (*S. mutans* UA159 and C180-2) were incubated on agar plates containing increasing concentrations of NaF (maximum of 52.6 mM). Colonies which could grow with high levels of fluoride were isolated and characterized. *S. mutans* strains were cultured anaerobically (80% N_2_, 10% CO_2_, and 10% H_2_) at 37°C using either Brain Heart Infusion (BHI) broth or agar.

### Growth of *S. mutans* UA159-FR With Fluoride

The growth of *S. mutans* UA159-FR and its parental strain UA159 was characterized on BHI agar and BHI broth with different levels of sodium fluoride (NaF). For growth assay on BHI agar, 5 μL of 10^1^ to 10^5^-fold serially diluted overnight cultures was spotted on BHI agar plates supplemented with 0, 2, 4, 6, 12, and 25 mM NaF. All plates were incubated anaerobically at 37°C for 3 days before recording images. For growth assay in BHI broth, overnight cultures of *S. mutans* UA159 and UA159-FR were diluted 1:20 in fresh BHI broth and grown until early exponential phase (OD_600_ ∼0.3). NaF was then supplemented to reach final concentration of 0, 5, 10, 20, 40, and 60 mM. All cultures were incubated at 37°C and the OD_600_ value was recorded every 30 min for a total duration of 20 h with SpectraMax Plus 384 (Molecular Devices, CA, United States). Both growth assay experiments were performed with triplicates.

### Genome Analysis and SIFT Prediction

**FIGURE 1:**
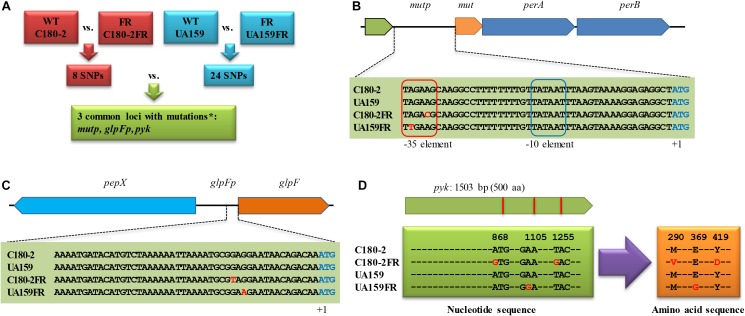
The identification of loci with mutations shared between *Streptococcus mutans* UA159-FR and C180-2FR. **(A)** Scheme for the comparison of the SNPs from the two fluoride-resistant *S. mutans* strains. The nucleotide sequence of loci with mutations shared in both fluoride-resistant *S. mutans* strains is shown for **(B)** *mutp*, **(C)** *glpFp*, and **(D)** *pyk*. WT, wild-type strain; FR, fluoride-resistant strain; SNP, single nucleotide polymorphism; ^∗^chromosomal regions in which mutations were found in both fluoride-resistant strains. *perA* and *perB* encode fluoride antiporters. *glpF* encodes a glycerol uptake facilitator protein. *pepX* encodes an x-prolyl-dipeptidyl aminopeptidase. *pyk* encodes pyruvate kinase. The nucleotides shown in red in **(B)** to **(D)** indicate identified SNPs. In **(D)**, the amino acid substitutions due to the SNPs in pyruvate kinase are also shown in red.

We compared the genome sequence of *S. mutans* UA159 (NCBI accession: NC_004350.2) to that of the fluoride-resistant strain *S. mutans* UA159-FR (NCBI accession: NZ_CP007016.1) to identify SNPs. Firstly, the genomes were aligned using progressive Mauve (version 2.4.0, snapshot February 26, 2015) (Darling et al., 2004, 2010). All SNPs provided by Mauve were manually examined to confirm whether they locate in a coding sequence (CDS) or an intergenic region. The identified non-synonymous and intergenic SNPs in *S. mutans* UA159-FR were compared to the published SNPs in *S. mutans* C180-2FR (Liao et al., 2015). Figure 1A shows the scheme for this comparison. The loci with mutations in both fluoride-resistant strains were selected for further investigation. Where relevant, the effect of the consequential amino-acid substitutions on the function of corresponding proteins was predicted with SIFT^1^ using default settings (Ng and Henikoff, 2003).

### Gene Expression in *S. mutans* UA159 and UA159-FR

Expression of the genes associated with mutations was examined in *S. mutans* UA159 and UA159-FR with real-time PCR. Both strains were grown in BHI broth until early exponential phase (OD_600_ = 0.2), late exponential phase (OD_600_ = 0.8) and stationary phase (average OD_600_ = 1.2). Samples were taken from each growth phase and RNA was extracted and processed as described previously (Liao et al., 2015). Primers used for tested genes are listed in Supplementary Table 1. *gyrA* and *recA* were used as reference genes (Smoot et al., 2001; Brenot et al., 2005). The 2^-ΔΔCt^ method was used for the log-transformed data to calculate relative gene expression (Liao et al., 2015). The experiment was performed in triplicate.

### Construction of Green Fluorescent Protein Reporter Strains and Green Fluorescence Assay

To evaluate the promoter activity alone, reporter strains were constructed for the selected promoter regions (*mutp*) obtained from the various *S. mutans* strains. Three *mutp* regions, including one wild-type *mutp* and two mutated *mutp* were used for the construction of the reporter strains. Only one wild-type *mutp* reporter construct was made because the *mutp* regions from *S. mutans* C180-2 and UA159 are identical. The *mutp* regions from *S. mutans* UA159, UA159-FR and C180-2FR were PCR-amplified with primer set *mutp*_gfp (Supplementary Table 1). The fragments were ligated into a shuttle vector containing a promoterless green fluorescent protein (GFP)-coding gene (pDM45) (Cormack et al., 1996). All constructs were separately transformed into *S. mutans* strain UA159. Later, the fluorescence intensities of overnight cultures of these reporter strains were quantified using the green fluorescence assay. The optical density of the cultures from the different strains was first adjusted to OD_600_ 1.0 by diluting with BHI broth. They were then diluted in PBS and measured with BD Accuri C6 Cytometer (BD Biosciences, CA, United States) using 488 nm as excitation wavelength and 530 nm as emission wavelength. Ten thousand events were collected in each sample. UA159 containing pDM45 was used as negative control. Data were acquired and analyzed with CFlow software (BD Biosciences, CA, United States). The experiment was performed in triplicate.

### Enzyme Activities

Since glycolytic enzymes were found to be associated with fluoride resistance of both strain UA159-FR and C180-2FR (see Results), the activities of pyruvate kinase and enolase were evaluated for both *S. mutans* pairs.

To examine the activity of pyruvate kinase, late exponential bacterial cells (OD_600_ = 0.8) were harvested with centrifugation, washed with 300 mM HEPES pH 7.0 (Sigma-Aldrich, St. Louis, MO, United States), and resuspended in the same buffer. The cells were then challenged with various concentrations (0, 2.5, 5, and 10 mM) of NaF in presence of 0.22 mM glucose. Milli-Q was added to the negative control group. After incubating at 37°C for 30 min, the cells were pelleted with centrifugation at 16,100 × g for 2 min. Cell-free extracts were prepared by suspending the pellets in lysis buffer (20 mM Tris–HCl pH 7.5, 0.04% Triton X-100 and complete protease inhibitor) and vigorously beating with 0.1 mm glass beads. The protein concentration in the cell-free extract was quantified using a Bradford protein assay kit (Life Science, California, CA, United States). Next, the cell-free extract was used for the pyruvate kinase assay (Yamada and Carlsson, 1975; Zoraghi et al., 2010). The reaction mix contained 60 mM Na^+^-HEPES pH 7.5, 100 mM KCl, 10 mM MgCl_2_, 2 mM ADP (Sigma-Aldrich), 0.24 mM NADH (Sigma-Aldrich), 0.5 mM glucose-6-phosphate (Sigma-Aldrich) and 12 μg/ml LDH (Sigma-Aldrich). Ten mM phosphoenolpyruvate (PEP) (Sigma-Aldrich) was added to the experimental group and the same volume of Milli-Q was added to the control group. The reaction was started by adding the 10-fold diluted cell-free extract to the reaction mix and followed by recording absorbance at 340 nm at 25°C for 40 min using the SPECTRAmax PLUS 384 (Molecular Devices Corp., Sunnyvale, CA, United States). The activity of pyruvate kinase was calculated as the amount of pyruvate produced per min per mg total protein. The experiment was repeated four times.

The activity of enolase was examined using a previously established protocol (van Loveren et al., 2008). Briefly, cells from mid-exponential phase (OD_600_ = 0.6) were permeabilized, incubated with 20 mM potassium phosphate buffer pH 7.0 containing 0, 0.5, 1, or 5 mM NaF for 30 min, and then used for the enolase assay. Enolase activity was measured by monitoring the formation of PEP at 240 nm (van Loveren et al., 2008). The experiments were repeated four times.

### Statistics

Data was analyzed with GraphPad Prism (version 5.00, GraphPad Software, San Diego California, CA, United States). Student’s *t*-test was performed for comparing the gene expression between two strains in each growth phase. The *p-*values from the comparisons were corrected for multiple testing using false discovery rate (FDR). One-way ANOVA and Turkey’s multiple comparisons test was performed to compare fluorescence intensities of three reporter strains. Two-way ANOVA was performed to compare the activities of enolase and pyruvate kinase under different fluoride concentrations in different strains. Differences were considered significant for *p* < 0.05.

## Results

*S. mutans* UA159-FR showed stronger fluoride resistance during growth when compared to *S. mutans* UA159. Bioinformatics analysis revealed that the *S. mutans* UA159-FR genome harbor a total of 24 SNPs. After comparing these SNPs with those found in *S. mutans* C180-2FR genome, we located three shared loci with mutations in both fluoride-resistant strains. Evaluation of these shared loci found changes in gene expression or alterations in protein functions.

### Characterization of Growth

**FIGURE 2:**
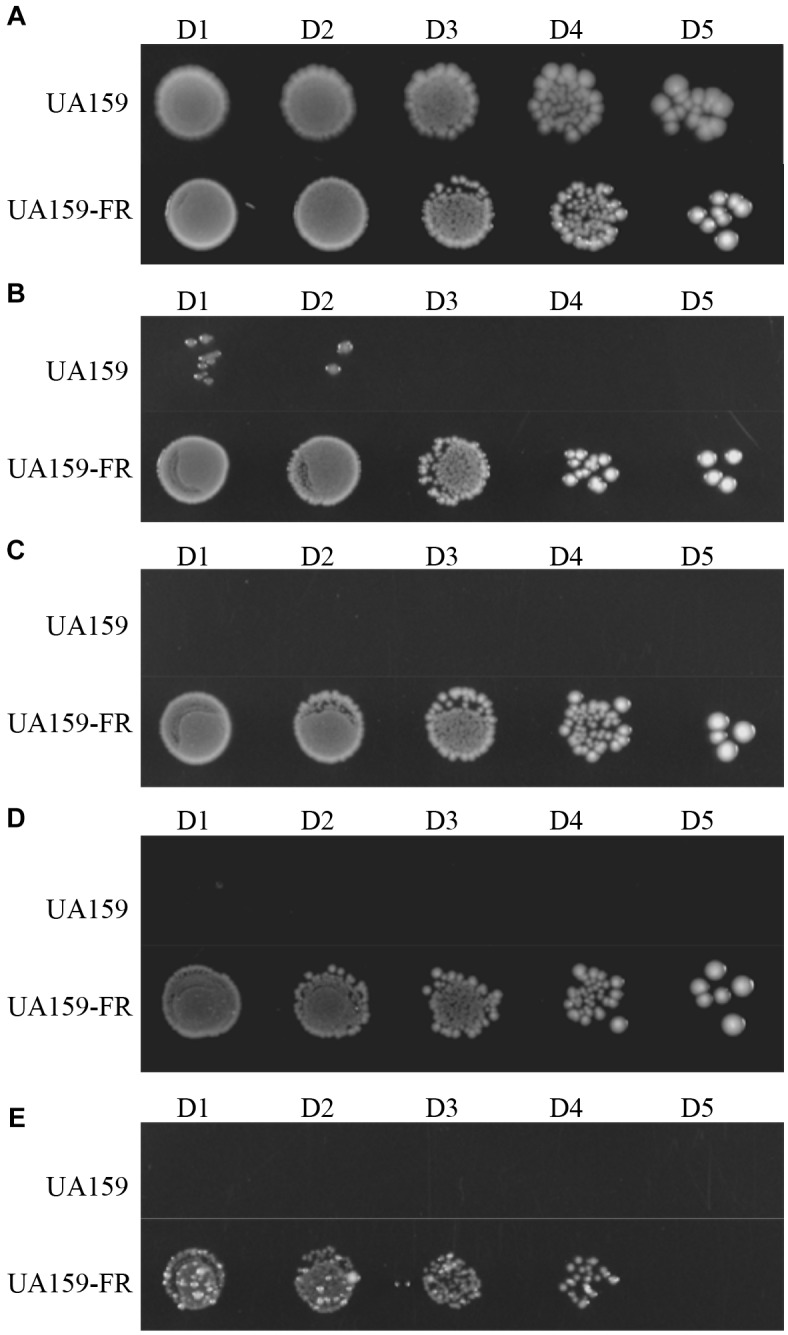
Representative growth of *S. mutans* UA159 and UA159-FR on BHI agar plates. NaF is supplemented at increasing concentrations: **(A)** without NaF; **(B)** 4 mM NaF; **(C)** 8 mM NaF; **(D)** 12 mM NaF; **(E)** 25 mM NaF. Overnight grown cultures were serially diluted 10^1^-fold (D1) to 10^5^-fold (D5).

In the absence of NaF, *S. mutans* UA159 had a significantly shorter doubling time (54.4 ± 1.7 min) than UA159-FR (65.4 ± 0.3 min) in BHI broth (*p* < 0.05, Supplementary Figure 1). The lag phase of UA159-FR was seemingly longer than that of UA159. On BHI agar, UA159-FR grew in the presence of higher fluoride concentration (25 mM) whereas the wild type UA159 stopped growing at 4 mM NaF, which confirms the fluoride resistance phenotype of UA159-FR (Figure 2). Similarly, in BHI broth, UA159-FR showed full growth with as much as 20 mM NaF while UA159 was obviously inhibited by the adding of only 5 mM NaF (Supplementary Figure 2). *S. mutans* C180-2FR, characterized in the previous study (Liao et al., 2015), also had a longer doubling time (in absence of NaF) and stronger ability to grow with NaF than its wild-type strain. It is worth mentioning that we tested growth inhibition instead of cell killing to evaluate fluoride resistance in *S. mutans*.

### Identification of Shared Loci and SIFT Prediction for Amino-Acid Substitutions

The comparison of the genomes of UA159 and UA159-FR identified 24 SNPs (Supplementary Table 2). This list was compared to that from C180-2FR, which was reported previously (Liao et al., 2015). Shared loci containing mutations in both fluoride-resistant strains were identified (Figure 1).

In total, three loci contained mutations in both fluoride-resistant strains. Two of them (*mutp* and *glpFp*) are intergenic regions and one (*pyk*) is a gene from the glycolytic pathway. The nucleotide or amino-acid sequences of these loci are shown in Figures 1B–D. Mutations in *mutp* from C180-2FR and UA159-FR were both located in the putative -35 element. Three downstream genes, encoding a putative mutase (*mut*) and two fluoride antiporters (*perA* and *perB*), are regulated by *mutp* (Figure 1B). The other shared mutation located in the intergenic region (*glpFp*), which is shared by two genes encoded in opposite directions. One gene encodes glycerol uptake facilitator protein (*glpF*) and the other gene encodes an x-prolyl-dipeptidyl aminopeptidase (*pepX*) (Figure 1C). C180-2FR had 2 SNPs in *pyk* which led to two amino-acid substitutions (M290V and Y419D), while UA159-FR had one SNP in *pyk* leading to one amino-acid substitution (E369G) (Figure 1D). Except the abovementioned loci, a SNP was identified in the enolase-coding gene (*eno*) of UA159-FR (Supplementary Table 2) This mutation resulted in an amino-acid substitution (T287I). Enolase catalyzes the reaction upstream of pyruvate kinase, namely the transformation from 2-phosphoglycerate to PEP. Like pyruvate kinase, it also plays an important role in glycolysis, and therefore was also examined.

SIFT prediction was performed for the amino-acid substitutions in pyruvate kinase and enolase. The Y419D substitution in pyruvate kinase of C180-2FR was predicted to be deleterious to the protein function (score 0.01). The other mutation, namely the E369G substitution, in pyruvate kinase of C180-2FR was predicted to be tolerated. The substitution in enolase of UA159-FR (T287I) was predicted to affect protein function (score 0.00) but with low confidence, as the reference sequences were reported to be not diverse enough.

### Gene Expression and Fluorescence Intensities of Reporter Strains

**FIGURE 3:**
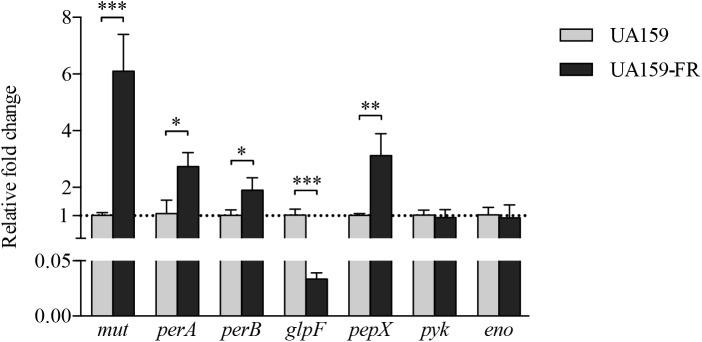
Relative fold change of gene expression between *S. mutans* UA159 and UA159-FR. Results from *S. mutans* UA159 and UA159-FR at late exponential phase are shown. Overall expression of each selected gene in UA159-FR relative to that in UA159 is presented as average fold change ± standard deviation. This experiment was repeated three times. All *p-*values were corrected for multiple testing. ^∗^*p* < 0.05; ^∗∗^*p* < 0.005; and ^∗∗∗^*p* < 0.0005.

The bioinformatics analysis uncovered three shared loci with mutations in the two fluoride-resistant strains. Thus, the expression of 7 genes closely related to the shared loci, *mut, perA, perB, glpF, pepX, pyk*, and *eno*, was examined in UA159 and UA159-FR. Mutations were identified either in the coding region or intergenic region of these selected genes. Figure 3 shows the relative gene expression of samples taken from late exponential phase. All three genes downstream of *mutp*, namely *mut, perA*, and *perB*, showed a significantly higher expression in UA159-FR than in UA159. The expression of *mut* in UA159-FR was 6-fold higher than in UA159 (*p* < 0.0005), while *perA* and *perB* showed approximately 2-fold up-regulation (*p* < 0.05). One of the downstream genes of *glpFp, glpF*, had a significantly lower expression (about 30-fold) in UA159-FR than in UA159 (*p* < 0.0005). The other downstream gene, *pepX*, showed a 3-fold higher expression in UA159-FR (*p* < 0.005). The other two tested genes, *pyk* and *eno*, did not show differential expression. The results of samples from early exponential and stationary phases were similar to those from late exponential phase (data not shown). The expression of these genes (except for *eno*) in C180-2 and C180-2FR was examined and reported previously (Liao et al., 2015). Briefly, the expression of *mut, perA* and *perB* in C180-2FR was approximately 10-fold higher than that in C180-2. The expression of *glpF* in C180-2FR was significantly lower (about 3-fold) than that in C180-2 in early exponential phase. The expression of *pepX* and *pyk* did not differ between C180-2 and C180-2FR.

The fluorescence intensities (FI) were quantified for the three *mutp* reporter strains (Supplementary Figure 3). UA159 containing *mutp* from either C180-2FR or UA159-FR showed significantly higher fluorescence than the reporter strain containing wild-type *mutp* (*p* < 0.0005). No statistically significant difference was found between the FI of the two fluoride-resistant *mutp* reporter strains.

### Activities of Pyruvate Kinase and Enolase

**FIGURE 4:**
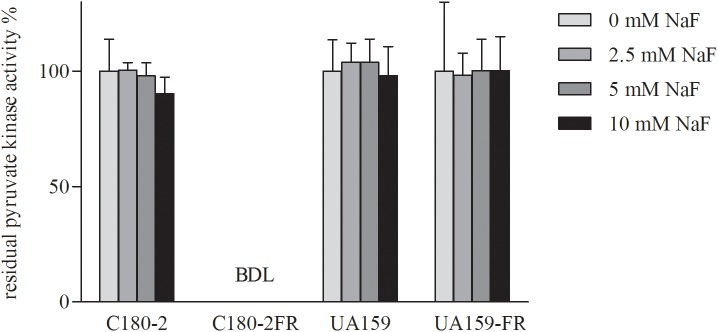
Residual activity of pyruvate kinase in *S. mutans* C180-2, C180-2FR, UA159, and UA159-FR after incubation with different concentrations of NaF. The activity of the control group which was incubated without NaF was set at 100%. BDL, below detection limit.

**FIGURE 5:**
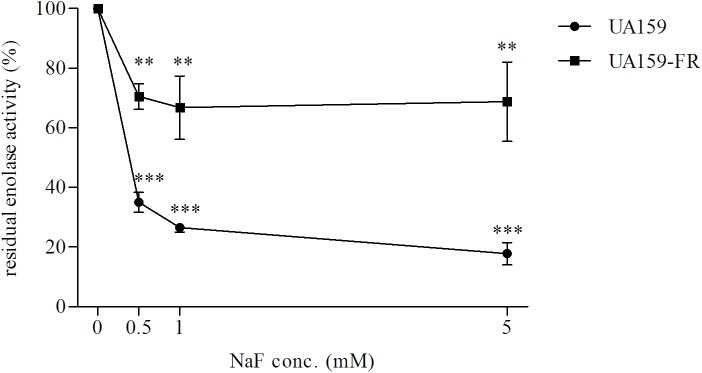
Residual enolase activity of *S. mutans* UA159 and UA159-FR after pre-incubation with different concentrations of NaF. The activity of the control group in which no NaF was added during pre-incubation was set at 100%. ^∗∗^*p* < 0.01 and ^∗∗∗^*p* < 0.001.

Since both in the literature (Belfiore et al., 1989) and in our pilot study, pyruvate kinase activity was found to be severely inhibited by high glucose concentrations, we used limited glucose (0.22 mM) in our experiment. UA159 and its fluoride-resistant derivative UA159-FR showed similar pyruvate kinase activities, with all tested concentrations of NaF (Figure 4). While C180-2 exhibited detectable pyruvate kinase activity under all conditions, C180-2FR showed no activity at all, independent of the presence of NaF (Figure 4). Enolase from both UA159 and UA159-FR was inhibited by NaF. The inhibition was stronger in UA159 than in UA159-FR (Figure 5). After pre-incubation with 0.5 mM NaF, only 35% of the original enolase activity remained in UA159 (*p* < 0.001), while approximately 70% activity was retained in UA159-FR (*p* < 0.01). Increased levels of NaF led to more inhibition in enolase from UA159, with only 18% residual activity after incubation with 5 mM NaF (*p* < 0.001). For UA159-FR, the inhibitory effect of 5 mM NaF is similar to that of 0.5 mM NaF. The enolase activity of C180-2 and C180-2FR has been evaluated in a previous study (van Loveren et al., 2008). Enolase from these two strains was similarly inhibited by NaF.

## Discussion

Bacteria can acquire resistance to fluoride through a mutation in the promoter of fluoride antiporters (Liao et al., 2016). While the clinical significance of fluoride resistance is not clear yet due to the lack of clinical isolation in the last decade, efforts have been made to understand the mechanism of fluoride resistance. Existing evidence (Brussock and Kral, 1987; Liao et al., 2015) indicated that this may not be the only route for a bacterial cell to become fluoride resistant. In this study, we found multiple SNPs in two different fluoride-resistant *S. mutans* strains. After comparing the SNPs, we proved that there is overlap of chromosomal regions with SNPs between the two fluoride-resistant strains. These SNPs can lead to alterations in gene expression and protein functions.

In the present study, we found mutations in two glycolytic enzymes, enolase and pyruvate kinase, in both fluoride-resistant strains, UA159-FR and C180-2FR. The involvement of the key enzyme enolase is not surprising. The inhibition of enolase by fluoride contributes to the antimicrobial action of fluoride (Guha-Chowdhury et al., 1997). This enzyme was proposed to be insensitive to fluoride in fluoride-resistant strains (Bunick and Kashket, 1981). However, most previous studies did not find either activity alteration or a mutation in enolase (Bunick and Kashket, 1981; van Loveren et al., 2008). In our study, we identified a mutation in the enolase gene of UA159-FR, which likely resulted in a profoundly decreased sensitivity of enolase to fluoride (Figure 5). This enables the bacteria to metabolize in the presence of high fluoride concentrations.

However, in C180-2FR, there is no mutation in the enolase gene. Instead, another glycolytic enzyme exhibited different behavior compared to the wild-type strain. The Y419D substitution in pyruvate kinase of C180-2FR resulted in a complete deactivation of this enzyme (Figures 1D, 4). Pyruvate kinase catalyzes the transformation from PEP to pyruvate, which is essential for subsequent acid production (Valentini et al., 2000). The malfunction of pyruvate kinase in C180-2FR can lead to drastic changes in glycolysis. In order to maintain pyruvate production, alternative pathways may be activated or up-regulated. One such pathway can be realized via an aminotransferase (YfbQ), which transfers an amine group from L-alanine to 2-oxoglutarate, with the concomitant production of pyruvate and glutamate (Ajdic et al., 2002; Yoneyama et al., 2011). More studies are needed to confirm the presence of alternative pathways in C180-2FR. Interestingly, we found malfunction of either enolase of pyruvate kinase (but not both of them) in UA159-FR and C180-2FR. We assume that the change of one of the two enzymes is profound enough to alter glycolysis, which is closely related to fluoride resistance.

Apart from the glycolysis pathway, we found two intergenic regions, namely *mutp* and *glpFp*, that harbor shared mutations in the two fluoride-resistant strains. Previous studies have shown that the deletion of the downstream genes of *mutp*, which encode fluoride antiporters, caused greatly increased fluoride sensitivity in wild-type *S. mutans* (Men et al., 2016; Murata and Hanada, 2016). In our recent study, we have confirmed the regulation of the fluoride antiporters through mutations in *mutp* (Liao et al., 2016). Indeed, the current result seems to support that this is an important and universal route to acquire fluoride resistance, as it exists in different fluoride-resistant strains. However, we noticed different levels of up-regulation in the two fluoride-resistant strains. UA159-FR expressed only two–threefold more *perA* and *perB* than its wild-type strain, while C180-2FR expressed 10-fold more *perA* and *perB* than its wild-type (Figure 3; Liao et al., 2015). In hope of explaining the difference, we compared the fluorescence of the reporter strains with *mutp* region from UA159-FR and C180-2FR. However, similar fluorescence intensities were found in the two reporter strains, indicating that the promoter activities of *mutp* from the two fluoride-resistant strains were similar (Supplementary Figure 3). The difference could due to the fact that the two SNPs in the two fluoride-resistant strains are two bases away from each other (Figure 1B), which has slightly different effects on the promoter function. More detailed studies on the promoter structure can help explain the different effects of the two mutations.

In contrast to the regulation of the fluoride antiporters, the knowledge on the function of the shared mutation in the second intergenic region (*glpFp*) in fluoride resistance is limited. Since this region harbors the promoters for *glpF* and *pepX*, it is not clear how exactly the function of these genes was affected by the mutation(s). Our data showed down-regulation of *glpF* in both fluoride-resistant strains when compared to the wild-type strains; while the expression of *pepX* was up-regulated in strain UA159FR and unchanged in C180-2FR (Figure 3; Liao et al., 2015). It seems that *glpF* was more likely to be involved in fluoride resistance than *pepX*. Nevertheless, unlike *mutp* whose promoter elements are clear, the -10/-35 elements of the promoters within *glpFp* are not clear yet, due to the different directions of the downstream genes *glpF* and *pepX*. We currently cannot relate the down-regulation of *glpF* directly to the promoter activity which might be altered due to the SNPs. The promoter structure as well as the exact function of these two genes in fluoride resistance requires further research, using for example, RACE-PCR technique (Haenni et al., 2007).

Our current data suggests fluoride resistance may be related to several chromosomal mutations, as assumed in a previous study (Brussock and Kral, 1987). We do not know if multiple chromosomal mutations contribute synergistically to fluoride resistance. A hint is given by comparing UA159-FR with a genetically engineered strain UF35 (Liao et al., 2016). Unlike UA159-FR, which has multiple mutations, UF35 only has one mutation in *mutp* which up-regulates the fluoride antiporters. UA159-FR is able to grow with much higher level of fluoride than UF35 on BHI agar (Liao et al., 2016), implying an additive effect of multiple SNPs.

## Conclusion

In conclusion, by comparing the genome sequences, we have identified overlap of chromosomal regions with SNPs in two different fluoride-resistant *S. mutans* strains. Validation of these regions, including two intergenic regions (*mutp* and *glpFp*) and one pathway (glycolysis), showed alterations in gene expression and protein functions. Our results provide novel candidate loci involved in fluoride resistance. The role and importance of these loci on gene regulation in presence or absence of fluoride will be subjects of follow-up studies.

## Author Contributions

YL, JL, WC, CvL, BB, and DD conceived the experiments. YL and JY conducted the experiments. YL and JY performed the data analysis. YL wrote the manuscript. YL, JL, WC, CvL, BB, and DD modified the manuscript.

## Conflict of Interest Statement

The authors declare that the research was conducted in the absence of any commercial or financial relationships that could be construed as a potential conflict of interest. The handling Editor declared a past collaboration with several of the authors BB, WC, and DD.

## Footnotes

1 http://sift.bii.a-star.edu.sg/
