# Supplementary material for: Genetic Loci Associated With Fluoride Resistance in Streptococcus mutans
Source: Front Microbiol. 2018 Dec 11;9:3093. doi: 10.3389/fmicb.2018.03093 (PMC6297193; doi:10.3389/fmicb.2018.03093)
Supplement: Supplementary file 1 [file Data_Sheet_1.docx]

**Supplementary Table 1.** Primers used in this study.

| **Gene name** | **Primer** | **Sequence(5'-3')** | **Purpose** |
| --- | --- | --- | --- |
| *eno* | Forward | CGGATATGATGTTCGTGAT | Real-time PCR |
|  | Reverse | ACCAAGAATAGCATTAGCA |  |
| *glpF* | Forward | GTTACCAGATACATTACCA | Real-time PCR |
|  | Reverse | TACTGCTCTACTCGTTAT |  |
| *mut* | Forward | ATGGTGGAGCGATATGTA | Real-time PCR |
|  | Reverse | TGTTTAGAAAGACGAATGACT |  |
| *perA* | Forward | TTACTGCTGCTGGTATGG | Real-time PCR |
|  | Reverse | TGCTGATAAGGTTAATACTGTTAG |  |
| *perB* | Forward | AGATGCTAATCCTTGGTA | Real-time PCR |
|  | Reverse | TATGGTCTTCCTCTTCAA |  |
| *pepX* | Forward | TATGGCTGACTGGACTAA | Real-time PCR |
|  | Reverse | TTCCGCAATAATGACCTTA |  |
| *pyk* | Forward | GGTGAAGATGGCTATTGG | Real-time PCR |
|  | Reverse | CATTGGCTCCTTCTGTAAT |  |
| *mutp_gfp* | Forward | CATATGAGCCTCTCCTTTTACTTAAA | Construction of reporter strains |
|  | Reverse | GCATGCACTGATATTACTGGCTATTTA |  |

**Supplementary Table 2.** Single nucleotide polymorphisms identified from the genomes of *S. mutans* UA159 and UA159‑FR

| **UA159** | | **UA159‑FR** | | **mutation type** | **Gene annotation** |
| --- | --- | --- | --- | --- | --- |
| **SNP base** | **Amino acid** | **SNP base** | **Amino acid** |  |  |
| G | V | T | F | non_syn^a^ | transcriptional regulator (SMU_112c) |
| T | F | C | S | non_syn | hypothetical protein (SMU_448) |
| C | S | G | *^b^ | non_syn | DNA-directed RNA polymerase subunit omega (*rpoZ*) |
| AA | N | CG | R | non_syn | glucosyltransferase-I (*gtfB*) |
| C | R | T | C | non_syn | GMP synthase (*guaA*) |
| C | T | T | I | non_syn | histidine kinase sensor CiaH (*ciaH*) |
| A | E | G | G | non_syn | pyruvate kinase (*pyk*) |
| C | T | T | I | non_syn | enolase (*eno*) |
| A | * | T | L | non_syn | hypothetical protein (SMU_1292c) |
| T | S | C | G | non_syn | transposase, ISSmu1 (SMU_565c) |
| A | S | G | P | non_syn | transposase, IS150-like (SMU_1370c) |
| G | P | A | L | non_syn | transposase, IS150-like (SMU_1370c) |
| A |  | G |  | intergenic region | Downstream^c^: SMU_t14 |
| C |  | T |  | intergenic region | Downstream: SMU_t14 |
| A |  | G |  | intergenic region | Downstream: hippurate hydrolase (SMU_318) |
| G |  | A |  | intergenic region | Downstream: hippurate hydrolase (SMU_318) |
| A |  | G |  | intergenic region | Downstream: hippurate hydrolase (SMU_318) |
| A |  | G |  | intergenic region | Downstream: hippurate hydrolase (SMU_318) |
| G |  | A |  | intergenic region | Downstream: glycerol uptake facilitator protein (*glpF*); x-prolyl-dipeptidyl aminopeptidase (*pepX*) |
| C |  | A |  | intergenic region | Downstream: Mg^2+^/citrate transporter (SMU_1013c); hypothetic protein (SMU_1014) |
| C |  | A |  | intergenic region | Upstream^d^: hypothetic proteins (SMU_1546 and SMU_1547c) |
| C |  | T |  | intergenic region | Downstream: transcriptional regulator (SMU_1647c) |
| C |  | T |  | intergenic region | Downstream: SMU_t42 |

a) nonsyn: non-synonymous coding SNP.

b) *: stop codon.

c) downstream: the gene / genes downstream the intergenic region with the SNP.

d) upstream: the gene / genes upstream the intergenic region with the SNP.

**Supplementary Figure 1.** Growth curve of *S. mutans* UA159 and UA159-FR in the absence of NaF in BHI broth. Mean optical density at 600 nm ± standard deviation is shown. This experiment was performed with triplicates.

**Supplementary Figure 2.** Growth of *S. mutans* (A) UA159 and (B) UA159-FR in BHI broth supplemented with 0, 5, 10, 20, 40, 60 mM NaF. Data are expressed as means ± standard deviation of triplicates.

**Supplementary Figure 3.** Fluorescence intensities of *mutp* reporter strains, *S. mutans* UA159 containing the wild-type *mutp*, *mutp* from C180‑2FR, or *mutp* from UA159‑FR. *** *p* < 0.0005.
